# A combined approach to data mining of textual and structured data to identify cancer-related targets

**DOI:** 10.1186/1471-2105-7-354

**Published:** 2006-07-20

**Authors:** Pavel Pospisil, Lakshmanan K Iyer, S James Adelstein, Amin I Kassis

**Affiliations:** 1 Harvard Medical School, Department of Radiology, 200 Longwood Avenue, Boston, Massachusetts, USA; 2 Bauer Center for Genomics Research, Harvard University, 7 Divinity Avenue, Cambridge, Massachusetts, USA

## Abstract

**Background:**

We present an effective, rapid, systematic data mining approach for identifying genes or proteins related to a particular interest. A selected combination of programs exploring PubMed abstracts, universal gene/protein databases (UniProt, InterPro, NCBI Entrez), and state-of-the-art pathway knowledge bases (LSGraph and Ingenuity Pathway Analysis) was assembled to distinguish enzymes with hydrolytic activities that are expressed in the extracellular space of cancer cells. Proteins were identified with respect to six types of cancer occurring in the prostate, breast, lung, colon, ovary, and pancreas.

**Results:**

The data mining method identified previously undetected targets. Our combined strategy applied to each cancer type identified a minimum of 375 proteins expressed within the extracellular space and/or attached to the plasma membrane. The method led to the recognition of human cancer-related hydrolases (on average, ~35 per cancer type), among which were prostatic acid phosphatase, prostate-specific antigen, and sulfatase 1.

**Conclusion:**

The combined data mining of several databases overcame many of the limitations of querying a single database and enabled the facile identification of gene products. In the case of cancer-related targets, it produced a list of putative extracellular, hydrolytic enzymes that merit additional study as candidates for cancer radioimaging and radiotherapy. The proposed data mining strategy is of a general nature and can be applied to other biological databases for understanding biological functions and diseases.

## Background

Recent advances in genomics and associated high throughput technologies have resulted in the exponential growth of biological databases. These consist of annotated genomic databases such as those at NCBI Genomic Biology [1], Ensembl [2] and UCSC Genome Bioinformatics [3]; specialized primary databases of proteins including UniProt (the universal protein resource) [4] and the RCSB Protein Data Bank (PDB, the database of protein structures) [5]; and derived databases such as EMBL-EBI InterPro (database of protein families, domains and functional sites) [6]. In parallel with structured data, the corpus of scientific literature (textual data) has been expanding rapidly. Structured and textual data are fertile grounds in the bioinformatics community for the development of data mining tools to identify key entities (genes/proteins) involved in biological processes and provide important biological insights. The combination of these two resources has resulted in knowledge bases that represent derived information on interactions among entities (see references [7-12]).

Deriving biological insights from these unprecedented quantities of data is a challenge [13-16]. In recent years, tools for data mining retrieval of information have made considerable progress [17-20]. However, several problems persist: (i) incorrect, incomplete or several (synonyms, homonyms etc.) descriptions of entities, (ii) inefficient search engines associated with databases, (iii) constant need for annotation updates and for query-tool improvements, and (iv) inherent complexity of the biological phenomena and biological systems [17,21,22]. Moreover, various databases and their associated tools provide information at different levels of reliability and accuracy [21].

**Figure 1:**
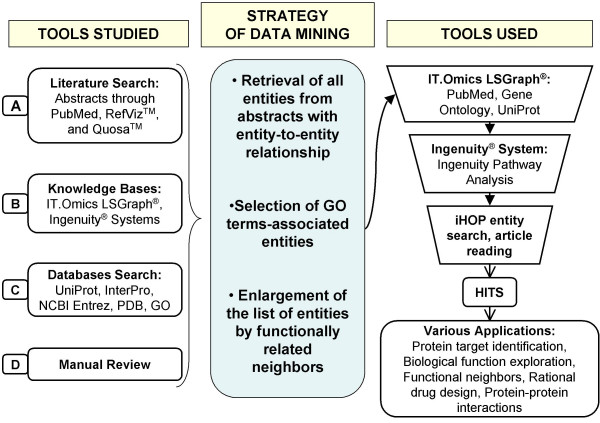
**Data mining tools and strategy**. Building effective data mining strategy and the sequence of programs used in the proposed strategy.

We present a systematic strategy for data mining that aims to address the above-mentioned problems (Figure 1). It is based on (i) use and combination of several resources (literature text, structured databases and knowledge bases), (ii) retrieval capabilities of text mining tools selected for this purpose, (iii) search within the Gene Ontology (GO) [23] and filtering of entities by GO terms within knowledge bases, and (iv) enlargement of the searched space by functionally-related entities. The strategy is conceived so that it can be used by any researcher for a particular problem. The intent is to develop a facile and reliable data mining approach that allows the identification of new targets.

To develop the appropriate strategy, we have explored and tested the performance of various data mining tools and databases to arrive at a protocol that is most appropriate for addressing our needs as well as those of users with other criteria. Specifically, our sequence of procedures examined PubMed abstracts [24], gene/protein databases – UniProt [4] and NCBI Entrez Gene [25], and state-of-the-art pathway knowledge bases – IT.Omics LSGraph^® ^[8] and Ingenuity^® ^Systems, Ingenuity Pathway Analysis [9].

The method was applied to the identification of cancer-related enzymes with hydrolytic activities that are overexpressed in the extracellular space of solid tumor cells. The developed strategy was evaluated by determination of hydrolases suitable for our in-house EMCIT (Enzyme-Mediated Cancer Imaging and Therapy) technology [26], which aims to precipitate water-soluble radioactive prodrugs within the extracellular spaces of solid human tumors for their diagnosis and/or therapy. In general, these proteins are attached to the membrane via glycopolyinositol (GPI) linkers or they are secreted in the extracellular space.

We have used the method to distinguish hydrolases that are overexpressed by human prostate, breast, lung, colon, ovarian, and pancreatic cancers. The approach has led to the recognition of a number of cancer-related putative targets for the EMCIT technology, including prostatic acid phosphatase (PAP), prostate-specific antigen (PSA), and sulfatase 1 (SULF1). Finally, the strategy and selected tools have helped us to determine several proteins that may be useful in other fields of cancer biology, specifically in the identification of biomarkers valuable in diagnosis, prognosis, and therapy [27-30].

## Results and discussion

### Strategy setting

The basic idea is the development of a series of data mining steps of increasing stringency such that the huge number of entities (genes/proteins) present in the literature and in various databases is reduced to a manageable size. At each step, the criteria, choice of tool, and databases are selected by the user to increase the list of identified hits. We employ LSGraph and IPA as suggested in this section; however, depending on the availability of tools or the focus of the user, other tools and databases can be "plugged in" to the proposed data mining strategy.

Our method using LSGraph [8] and Ingenuity Pathway Analysis (IPA) [9] applications (complemented by GO and PubMed) is founded on the combination of selected tools and the comparison of the identified resulting hit list with that provided by independent searches of the literature (**A**), knowledge bases (**B**), gene/protein databases (**C**), and retrieval of information from the text by the reader (manual review, **D**) (Figure 1). The method provides overall the largest list of proteins with desired properties.

The function of a protein is described in most detail in the scientific publications. However, due to the condensed nature of abstracts, scarce annotations in gene/protein databases, and the complexity of biological systems, this information may be difficult to acquire and to integrate. Therefore the first step in our strategy is based on the selection of the tool that, if possible, recognizes term variations within the text (in the full-text article preferably) and has the capability to retrieve entities from the text and export them to the list (here using LSGraph, PubMed).

Since we are mining for "unknown" proteins within a particular cellular location (extracellular, cell surface, plasma membrane, GPI-anchored), the second step of the strategy uses Gene Ontology as a tool in the process (using LSGraph). The inclusion of filtering by Gene Ontology organizing principles will allow any user to identify new targets for a particular problem. This can be applied to any search within the context of biological process, molecular function, or cellular component.

A protein may be implicated in a function or a disease because of its ability to interact with another protein that is already known in that function or disease. A straightforward search for proteins concerned with a specific function may not reveal all those involved. The third step of the present strategy includes the enlargement of the hit list to functionally related neighbors, which may compensate for the missing information and unmask the so-far-unrevealed protein function. In our particular case, this would ascertain the identification of entities involved in cancer. Therefore, we have extended the search to functionally related genes and proteins (using LSGraph and IPA).

### Selection of tools and databases

The performance of the combined searches is compared with that employing exclusively either a single literature or a database data mining method. The query of PubMed only (method **A**), with subsequent retrieval and reading of full texts one-by-one (method **D**) and the identification of desirable entities, is time- and labor-intensive. The search using Thomson RefViz™ [31] and Quosa™ [32] (see Methods) leads to the clear interconnection of abstracts and limits the number of publications to be studied. These programs, however, cannot be used to extract the assignment of names, synonyms, and symbols to their appropriate entities (genes/proteins) and to save them as a set; thus, the list of entities of interest cannot be easily and routinely generated. In contrast, LSGraph has literature and entity retrieval capabilities that make it a suitable tool.

Method **C **also has its shortcomings. Generally, databases provide their own query tools that may produce biased results. The problem consists of (i) incomplete or misleading protein annotations, (ii) unsatisfactory query-engine capabilities of protein databases, and (iii) unclear or incomplete functional description. Retrieved results are often specific database limited: databases differ from each other and are not updated regularly or their updates are not synchronized. Searching for functionally related proteins is sometimes not possible. In comparison with the combined data mining approach, protein-database search is inefficient (even though these databases represent the largest protein information depositories). For example, the query for prostate extracellular protein results in only 3 to 5 hits (depending on the depository searched), whereas our novel strategy produces 135 hits (Table 1).

Method **B **employs the LSGraph and Ingenuity Pathway Analysis (IPA) pathway knowledge bases. We have combined the advantages of both of these applications in order to optimize the yield of the data mining process. Moreover, they have the capability to import and visualize the expression-fold of genes from microarray experiments.

The essential power of LSGraph is its ability to identify all relevant entities in all PubMed abstract citations («Most cited entities» function, see Methods) and to cluster them subsequently into a set. LSGraph incorporates the system of functional and process-oriented annotations, based on the Gene Ontology (GO), which allows selecting the entities associated with GO terms «extracellular region» or «membrane». Both Gene Ontology terms have been selected because they deliver the highest number of relevant hits. Although the results include transmembrane- and mitochondrial-membrane-associated proteins, these can be removed later by limiting the search to a desired subcellular location in IPA.

**Figure 2:**
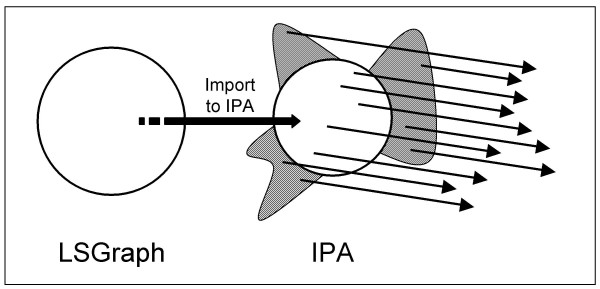
**Transfer of entities between two knowledge bases**. For LSGraph, PubMed and GO, all extracellular or membrane-bound entities cited in scientific abstracts were exported using UniProt accession numbers into IPA (thick arrow) for each cancer type. For IPA, imported entities were recognized and enlarged by functional neighbors (shaded area) of «Global Analysis Genes» network. All entities with «High Level Function» corresponding to «cancer» (thin arrows) were exported to Microsoft Excel workbook as hit lists for further individual examination (see Additional file 1).

Ingenuity Pathway Analysis [9] is an application built on one of the largest knowledge bases (Ingenuity Pathway Knowledge Base), acquired by manual curation of full texts of peer-reviewed scientific publications. It covers information on more than 23,900 mammalian genes and thousands of corresponding proteins and their relations (1.4 million biological findings, December 2005) [9]. Enlargement of the set of extracellular entities (defined by LSGraph) by functional neighbors of the IPA knowledge base and subsequent focusing on entities discussed in the context of cancer have enabled us to select entities suitable as targets for EMCIT drug design (Figure 2). Both LSGraph and IPA contain information about human, mouse and rat species. If the mining is required for other species, e.g. plants or yeast, it is possible to plug into the strategy discussed above the appropriate tools and databases. Hence, the strategy will remain essentially identical and, within the scope of selected tools, is generic in nature; we can address various biological problems, for example, investigate the genes involved in aggregation of proteins in Alzheimer disease or identify key players and novel targets in pathways involved in this disease.

### Cancer-related data mining case

The combined strategy using LSGraph and IPA (including PubMed, GO, and UniProt) (Figure 1) has led to the findings summarized as numbers of abstracts and proteins in Table 1, these being acquired in a period of a few weeks. The search for hydrolytic enzymes has been performed for six common tumor tissues (prostate, breast, lung, colon, ovary, and pancreas). First, a PubMed query has yielded between 90,000 to more than 640,000 articles per specific cancer-tissue type. Using the «filter» function in LSGraph reduces this number to between 11,000 and 42,000 abstracts containing sentences that describe entity-to-entity functional relations. The next step, which involves sifting for the keywords «extracellular» or «membrane», decreases these numbers to approximately 1,784 to 8,869 entities implicated in the extracellular environment, which corresponds to about 1,791 to 4,325 proteins. Further filtering by the Gene Ontology database in LSGraph annotates 749 to 1,718 proteins as extracellular or membrane-bound.

**Figure 3:**
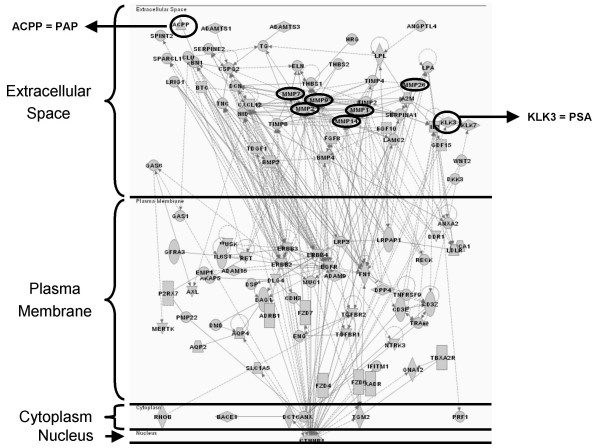
**Example of network of prostate-cancer-related proteins**. This network was generated through the use of Ingenuity Pathway Analysis (IPA). Gene products are represented as nodes and biological relationships between two nodes as a line. Shapes of nodes symbolize functional class of gene product; for example, triangles are phosphatases, diamond-shaped rectangles are peptidases. Proteins are separated in spaces between lines corresponding to cellular location based on IPA-location categories: Extracellular space, Plasma membrane, Cytoplasm, and Nucleus. Some proteins of interest are circled: prostatic acid phosphatase (PAP) is coded as ACPP, prostate-specific antigen (PSA) as KLK3 (kallikrein 3), and various metalloproteinases as MMP.

Over 375 enzymes, receptor domains, growth factors and other proteins have been found in the context of the six tumor-tissue types linked to «cancer». Using the IPA, 375 to 598 of these proteins are found to be within the «cancer» network (Table 1). The majority of the identified extracellular proteins for all tumor types are growth factors, tumor necrosis factors [33], and hydrolytic enzymes. The latter are placed within "Enzymes," "Peptidases," "Phosphatases," and other IPA families (Table 1). Further reading of the cited articles, retrospectively tracked by iHOP [34], has enabled us to select those that are appropriate for our in-house EMCIT concept. The list of hits is enclosed with this article on-line, see Additional file 1. From this final dataset, we have identified several hydrolases, among them peptidases (matrix metalloproteinases and kallikreins) and phosphatases (see Figure 3 for IPA subnetwork) and several known cancer progression markers, matrix metalloproteinases (MMP1, MMP2, MMP3, MMP7, MMP9) [35] and coagulation factors II, III, XII (Hageman factor, F11), located at the extracellular surface [35,36]. Another potential target retrieved in all six cancer types is cathepsin B, a hydrolase expressed in brain tumors and suggested as an agent for the imaging of tumor progression [33].

There are also apparent pitfalls/shortcomings in the knowledge bases. In IPA, for instance, the cellular component annotation is sometimes missing or incomplete due to the lack of information in protein databases to which IPA is ultimately linked (e.g. UniProt, Pfam [37], Entrez Gene). In the case of cellular location, this could mean that the set of extracellular entities may be an underestimate and that the 135 hits found, for example, in prostate cancer may be only a subset. Links to curated databases are valuable; however, the major knowledge is still hidden in the full text of the scientific literature.

### Identification of promising targets

#### Prostatic acid phosphatase

The success of our strategy can be demonstrated in the identification of PAP (for our purposes as an EMCIT candidate). In UniProt, the prostatic acid phosphatase (PAP, gene name ACPP) is not found when three keywords are used: "prostate", "acid" and "phosphatase." This problem has a crucial impact when searching through databases. Generally, restricting the scan to annotation fields of databases (UniProt or Entrez Gene [25]) is faster but doing so can provide an outcome that is limited by the *ad hoc *relationship of keywords to the research interests of whoever submitted the entry. Analogically, we observe similar problems when using NCBI protein or nucleotide databases, InterPro, PDB, OMIM™ (Online Mendelian Inheritance in Man, database of human genes and genetic disorders) [38] and Pfam (protein families database). Moreover keeping annotations current is difficult as new information appears frequently and regularly in the literature. When searching for proteins with specific properties without the knowledge of their actual name, symbol, or database identifier, data mining has to be performed with care.

Another issue with PAP is its cellular localization. While this hydrolase possessing protein tyrosine phosphatase activity has been demonstrated *in vitro *to dephosphorylate the epidermal growth factor (EGF) receptor present at an intracellular site in prostate carcinoma cells, PAP is also known to be secreted by epithelial cells in the prostate gland and found in seminal fluid (see references in [39]). UniProt does not specify the location of PAP; Entrez Gene provides the GO term "extracellular region" associated to PAP with a note that there is no record of it (NR, no record). It is safe to assume that since PAP is secreted outside cells, it will be localized in the extracellular space. Our recent *in vitro *cell analysis using noninternalizable PAP substrates shows that phosphorolysis occurs in the extracellular region (unpublished results). The combined data mining strategy ensures that PAP is not lost with the filtering of the results. It is identified as "ACPP – acid phosphatase, prostate" in LSGraph and situated by IPA in the «cancer» network (Figure 3). Both LSGraph and IPA document PAP as extracellular and, therefore, PAP has been identified and passed through the data mining step to the final list.

#### Alkaline phosphatase

Alkaline phosphatase (ALP) exemplifies another aspect of the annotation problem that is addressed uniquely by our strategy. This enzyme, known to be overexpressed at the surface of various solid tumors [40-45], is one of the first proteins identified as being expressed on the surface of cancer cells [46]. Many clinical observations have been made on the use of ALP as a tumor marker [47-50]. Searches for alkaline phosphatase in gene and protein databases have identified 19 protein sequences in humans, out of which only four are well-described: placental (gene name ALPP), placental-like (gene name ALPPL2), intestinal (gene name ALPI) and nonspecific tissue (liver/bone/kidney) (gene name ALPL). Databases state that human ALP is bound to the membrane by a GPI-anchor in contrast to mouse isoenzymes which are «extracellular». This illustrates, retrospectively, why we have not used the species specification in data mining. In the case of ALPI, UniProt also registers the GO term «membrane». For other ALP, the subcellular location is not present in annotations (noted "none"). Curiously, a similar situation occurs with Entrez Gene registrations of ALPP, ALPI and ALPL [Entrez Gene: 250, 248 and 249, respectively]. Due to this fact, the description of proteins as "attached to the membrane by a GPI-anchor" does not access them if searched for with "extracellular."

Specification of ALP searches in databases by the keyword "cancer" or by other neoplasm-related terms does not identify any of the above isoenzymes. UniProt annotation states that the exact physiological function of the alkaline phosphatases is not known [UniProt:PPB1_HUMAN]. Surprisingly, neither UniProt nor Entrez Gene (both of which are large depositories of information) states that these enzymes are related to cancer. In our data mining findings, alkaline phosphatase is listed in LSGraph under different isoenzymes (ALPP, ALPI etc.) in all six tissue types studied. However, ALP is subsequently lost within the IPA step of the data mining process due to missing annotation in the original gene/protein databases as well as its being placed within the IPA «cell-cell interactions» network and not the «cancer» network. Therefore, unless entries are thoroughly investigated one by one, a search using simple keywords in gene or protein databases will not identify alkaline phosphatase as an extracellular cancer-related enzyme.

#### Extracellular sulfatase 1

One of the new hits and a potential EMCIT target identified by the present strategy is a human enzyme, extracellular sulfatase 1 (SULF1) [51-53]. This enzyme is situated in the endoplasmatic reticulum and Golgi stack and is also shown, by its similarity to homologous proteins, to be secreted outside the cell (as described in [UniProt:SULF1_HUMAN]. Although SULF1 contains the word "extracellular" in its protein name (Extracellular sulfatase 1 precursor), protein databases do not track a traceable author statement or publication with reference to its being an extracellular enzyme. Only Entrez Gene [Entrez Gene:23213] states that it is extracellular, referring to Morimoto-Tomita *et al*. [51]. Similar to the case with PAP, we have recently also demonstrated the hydrolysis of a noninternalizable sulfatase substrate by human pancreatic tumor cells, thereby ascertaining the presence of SULF1 in the extracellular space (unpublished results). Once more this demonstrates the idiosyncrasies of such searches since the use of database-only data mining (method **B**) would not have identified this sulfatase whose recognition within our hit list is consequent to the utilization of a combination of programs and the search within the space of functionally-related proteins.

#### Prostate-specific antigen

Another EMCIT candidate that has been established is prostate-specific antigen (PSA), a well-known and clinically useful prostate cancer marker that has proteolytic activities. This molecule, also known as kallikrein 3 (KLK3), is secreted in the extracellular space (Figure 3) by normal and malignant prostate cells [54,55]. Since this enzymatically active molecule is inactivated by binding to several protease inhibitors once it enters the circulation [54,56,57], we and others [58] have recognized its potential for targeting PSA-activated prodrug molecules to prostate cancer. PSA (under the name KLK3) has passed through the data mining and was identified in all six cancer types (Figure 3, Additional file 1).

As mentioned above, our interest in the identification of extracellular hydrolytic enzymes, such as prostatic acid phosphatase, alkaline phosphatase, sulfatase 1 and prostate-specific antigen, is driven by our focus on finding novel, radiolabeled molecules that are excellent substrates for such enzyme candidates, as these could be used to hydrolyze the radiolabeled prodrugs for our EMCIT approach. These enzymes can be further studied by *in silico *modeling simulations of their complexes with putative drugs. The prerequisite for molecular modeling of protein-ligand complexes is the availability of the three-dimensional (3D) structure for the target in the Protein Data Bank (PDB) or, in its absence, the building of a 3D-structure homology model. In our case, when these targets were checked for 3D-structure availability in the PDB, it was found that both PAP and ALP have already been crystallized (PDB codes 1ND5 and 1EW2, respectively) whereas the structures of human PSA (in contrast to that of the horse homologue) and SULF1 have not yet been solved.

## Conclusion

We have developed a new, rapid, data mining strategy that combines the literature, web-based gene-product databases, and complex knowledge pathway databases. The strategy, which is based on a combination of curated knowledge pathway bases with protein databases, has revealed the unique characteristics of several programs, including the entity retrieval capability and the Gene Ontology term-filtering specification of LSGraph and the full-text-based knowledge of IPA. The complementarity of content and functionalities of the query tools in the knowledge bases help to ascertain the identified hits as well as to discover other related neighbors of the known targets. We believe that this strategy overcomes many data mining drawbacks.

The application of this strategy to the search for extracellular hydrolases involved in human cancers has led to the discovery and identification of several previously "hidden" proteins that would not have been detected by a search using a single information source. Our findings indicate that the approach benefits from the complementarity of the databases and query tools employed and can distinguish useful and previously uncharacterized cancer-related targets with additional characteristics (other cellular localization, biological function) valuable in the noninvasive radiodiagnosis and treatment of cancer. Alkaline phosphatase (various cancers), prostatic acid phosphatase and prostate-specific antigen (prostate cancer), and extracellular sulfatase 1 (pancreatic cancer) are four interesting results. While these are being used in our laboratory as potential targets in the design of novel, radiolabeled diagnostic and therapeutic agents for cancer, the strategy for data mining is of a general nature and can be applied to other biological databases for understanding biological functions and diseases.

## Methods

A graphical representation including the steps of the strategy of our data mining method is presented in Figure 1 and Table 1. The sequence of tools used in our strategy has been determined after exploration of available tools and databases (that were accessible to us) in four different analysis components (Figure 1): literature databases (**A**), knowledge pathway databases (**B**), annotated gene/protein databases (**C**), and manual review (**D**).

### Literature (A)

The literature search (**A**) was done using PubMed and the RefViz™ (version 1.1.0) [31] and Quosa™ (version 7.14) [32] applications. RefViz and Quosa hunt through PubMed [24], Web of Science^® ^[59], and other literature sources and use symbolical, graphical visualization to explore the reference collections for the specific keyword.

### Knowledge bases (B)

The functional relationship, knowledge-base search (**B**) used the pathway analysis programs LSGraph (version 1.5, Life Science Graph^®^, IT.Omics, Lille, France) [8] and Ingenuity Pathways Analysis (IPA) (version 2.0, Ingenuity^® ^Systems, Mountain View, California, U.S.A.) [9]. LSGraph is free for academia and IPA provides (as do other similar commercial programs) free trials. Exploration was carried out for proteins with required characteristics (extracellular or integral to membrane, related to cancer). Both applications were used to enlarge the set of entities by functionally related neighbors (Figure 2). For both LSGraph and IPA, every entity functional relationship in the networks is supported by published information.

#### LSGraph and Gene Ontology

This application performs advanced bibliographic queries in the abstracts of PubMed by desired keyword («filter» function) and carries out exhaustive searches due to the systematic coverage of synonyms and homonyms. LSGraph can also interpret large-scale experimental datasets (microarrays). Its «Most cited entities» function allows the retrieval of the entities that are discussed most frequently in abstracts selected by «filter» function keyword and exports them into the user-defined set (see in detailed algorithm steps below). In our case, we set this function so that we retrieved *all *entities in the current literature, ranked in order from most to least cited.

Additional LSGraph function «Best GO» of the Gene Ontology can identify and rank all GO terms associated with the entities and subsequently downsize the list based on the GO term of choice. The Gene Ontology (GO) project is a collaborative effort that aims to address the need for consistent descriptions of gene products in different databases [60]. The GO consortium is developing three, structured, controlled vocabularies (ontologies) that describe gene products in terms of their associated biological processes, molecular functions, and cellular components in a species-independent manner. We used one of the organizing principles, cellular component, to associate the searched gene products annotated with the term «extracellular region» or «membrane». The «extracellular region» term ([GO:0005576]; 4434 entities share this term, December 2005) is hierarchically inferior directly under the cellular component tree but does not include proteins attached to the cell membrane. The term «membrane» ([GO:0016020]; 17193 entities share this term, December 2005) is attributed to proteins attached or within the membranes enclosing cells or any membranes of eukaryotic organelles. Later strategy-filtering with IPA-location categories can distinguish the plasma membrane specifically.

#### Ingenuity Pathway Analysis (IPA)

This is a web-based software application that allows users to identify the biological mechanisms, pathways, and functions most relevant to a dataset of genes or proteins of interest. It is founded on the largest knowledge base of biological networks created from millions of expert-curated relationships between genes, proteins, cells, tissues, drugs, and diseases. The networks linked to cancer are generated from the full text of articles (contrary to LSGraph) published in the most important scientific journals. Imported gene identifiers (here from LSGraph) are mapped to its corresponding gene object in the Ingenuity Pathways Knowledge Base (IPKB). These genes, called focus genes, are overlaid onto a global molecular network developed from information contained in the IPKB. Networks of these focus genes are then algorithmically generated based on their connectivity. The Functional Analysis of IPA identifies the biological functions and/or diseases that are most significant to the data set (e.g. cancer).

### Databases (C)

The gene/protein database search (**C**) was performed using the world's largest collections of genes and proteins, UniProt [4], InterPro [6] and NCBI Entrez [61]. UniProt (the Universal Protein Resource) is the catalog of information on proteins created by joining the information contained in Swiss-Prot, TrEMBL, and PIR. InterPro is a derived database of protein families, domains, and functional sites in which identifiable features found in known proteins can be applied to unknown protein sequences. It contains the most comprehensive and complete annotations of proteins in various organisms. NCBI Entrez (The Entrez Global Query Cross-Database Search System) searches simultaneously against a set of databases for proteins, genes, structures, conserved domains, homologues and other criteria. These three databases were selected based on our own experience, the content quantity and quality, and the need to ensure that we maximally covered the collections of gene products.

### Manual review (D)

Manual review of the literature (**D**) was performed on the full text of publications identified in PubMed queries directly or indirectly through online links to references in gene/protein databases.

### Tools applied in our strategy

The combined strategy suggested in this work is conceived as follows (Figure 1): use of an effective tool for entity retrieval from the literature text and gene/protein databases (LSGraph search in PubMed), selection (filter-down) of entities upon the specific user-defined GO terms, and subsequent enlargement of the entity list by functional pathway neighbors (LSGraph and IPA). The accuracy of the results was assessed using control proteins that we know are extracellular and involved in cancer.

The comparison of data mining components **A **though **D **led to the use of tools in the following sequence of steps (Figure 1, Table 1: first column):

(i) Using analysis tools incorporated in LSGraph, we filtered the complete corpus of the PubMed down to abstracts that contain evidences of entity-to-entity relationship and are related to one of the six tumor-tissue types. The keywords "cancer" or "tumor" were not used in the query, but later in IPA (see (v)), because the evidence for the disease at this level of the search may not necessarily be known. Instead, the tissue names, prostate/prostatic, breast, lung/pulmonary, ovary/ovarian, colon/colonic, pancreas/pancreatic, which are present in the abstracts, were used in the searches. To ensure that we would not miss studies on model organisms such as the mouse, the search was not limited to humans.

(ii) The LSGraph function «Most cited entities» was used to extract all entities present in retrieved abstracts defined by the «filter» keyword and order them from the most cited to the least cited entity.

(iii) Entities with localization specified by cellular component, «extracellular region», «membrane», of the Gene Ontology database (LSGraph «Best GO») were saved in a new set containing protein identifiers, *viz*. UniProt accession numbers.

(iv) The set with all UniProt identifiers was imported into the IPA and thus enlarged by related entities (functional neighbors) (Figure 2).

(v) The functional analysis of IPA can identify the biological functions and/or diseases that are most significant to the dataset. Genes from the dataset found to be associated with "cancer" were considered for further analysis (Figure 2). Up to this point, the filtering was done for tissue type only (prostate, breast, *etc*.) and not for the pathological process of interest, *i.e*. cancer. From this point on, the entities were sifted down to the set known to be involved in cancer by selecting the IPA subnetworks in which cancer occurs as the most significant annotation. Figure 3 is an example of subnetworks for prostate cancer.

(vi) The final set of filtered entities was exported and stored in a Microsoft Excel spreadsheet (see Additional file 1) containing the gene product name, synonyms, description, UniProt accession number (AC), IPA-defined cellular localization and protein family.

(vii) The identified extracellular proteins that were retained by the above-mentioned criteria were filtered by IPA protein family (defined by Entrez Gene). The final count of hits was compared with the number of hits identified by the literature search method (**A**) and the single gene/protein database method (**C**).

Detailed analysis of resulting enzyme hits was done retrospectively in their most cited articles using iHOP (information hyperlinked over proteins) [34], a program that finds links to gene and protein databases (UniProt, PDB and NCBI databases) and identifies the particular gene product if the gene name or synonym name is known.

The proposed method is rapid, and the protocol can be performed within several days. Steps (i) – (iii) are automatized searches where only the filtering keyword entries or GO term entries are required. Steps (iii) – (v) as well as step (vii) require the manual export/import of the UniProt identifiers from LSGraph into IPA and from IPA into Microsoft Excel file format. Resulting proteins hits with enzymatic activities were confirmed manually by checking the associated literature references; hydrolases were confirmed among "Enzymes," "Peptidases," "Phosphatases," and other IPA families. Particular cases (see Results and Discussion) were consulted in original publications.

## Authors' contributions

PP carried out the data mining work and drafted the manuscript. LKI conceived the study design, did preliminary data mining work, and helped to draft the manuscript. LKI and AIK initiated the work. AIK together with SJA participated in the design and coordination of the work, as well as interpretation of experimental *in vitro *results. All authors read and approved the final manuscript.

## Supplementary Material

Additional File 1 **Data mining hit list for six common cancer types**. This file contains detail information about gene products identified in six tumor-tissue types. The genes are listed in six separate Excel spreadsheets by gene name, synonyms, description, UniProt accession codes, and IPA-based cellular location and family. Click here for file

## Figures and Tables

**Table 1:** Number of enzyme hits identified in six common cancer types using the combined data mining strategy.^*a*^

**Tumor type**	**Prostate**	**Breast**	**Lung**	**Colon**	**Ovary**	**Pancreas**
**PubMed**: Abstracts about tissue types	*89839*^*b*^	*20393*8	*644380*	*133660*	*147373*	*164890*
**LSGraph**: Abstracts with entity-to-entity functional relations in different tumor-tissue types (function «filter»)	*10857*	*23875*	*42962*	*15808*	*24758*	*28356*
**LSGraph**: Abstracts remaining after filtering for keywords «extracellular» or «membrane»	*1784*	*3873*	*8869*	*4815*	*4683*	*5305*
**LSGraph**: All cited entities retrieved from filtered abstracts (function «Most cited entities»)	1791^*c*^	2500	4325	3030	3365	3059
**LSGraph**: Entities filtered additionally by Gene Ontology (function «Best GO») with the cellular component «extracellular region» or «membrane». Exported to IPA through UniProt accession numbers	749	941	1718	1186	1427	1244
**IPA**: Imported entities overlaid onto a global molecular network of IPA and enlarged by functionally related entities	917	1117	1954	1408	1675	1495
**IPA**: Entities within the network «cancer»^*d*^	375	456	598	481	515	450
**IPA**-location: Extracellular space	135	170	212	176	184	170
**IPA**-location: Plasma membrane	188	220	298	238	262	219
**IPA**-location: Extracellular space and Plasma membrane	323	390	510	414	446	389
**IPA**-family: Enzymes	12	14	20	17	16	16
**IPA**-family: Peptidases	24	25	33	24	30	31
**IPA**-family: Phosphatases	5	9	10	7	8	5
Other **IPA**-families^*e*^	282	342	447	366	392	337

^*a*^Completed on 11/18/05; ^*b*^in italic: number of abstracts; ^*c*^in normal font: number of entities (genes and proteins); ^*d*^numbers below correspond to subnetworks of entities designated by IPA-location or IPA-family and are all part of the IPA cancer network; ^*e*^includes some enzymes, for example human sulfatase 1
